# Single ventricle, bicuspid aorta and interatrial wall aneurysm as a rare complex adult congenital heart disease: a case report

**DOI:** 10.1186/1757-1626-2-109

**Published:** 2009-01-31

**Authors:** Blerim Berisha, Xhevdet Krasniqi, Agim Thaqi, Masar Gashi, Dardan Koçinaj

**Affiliations:** 1 Cardiology Department, Internal Clinic, University Clinical Center of Kosova, 10000 Prishtina, Republic of Kosova; 2 Coronary Care Unit, Internal Clinic, University Clinical Center of Kosova, 10000 Prishtina, Republic of Kosova

## Abstract

**Background:**

Single ventricle, bicuspid aortic valve and interatrial wall aneurysm in adulthood are a rare and unique case in medical literature. This presented case with congenital heart disease has never been treated surgically and clinical consequences seriously presented in adulthood.

**Case presentation:**

A 27 year old man with complex congenital heart disease presented. At the age of six, the single ventricle was ultrasonographly diagnosed, but at age 27 clinical consequences started to be seriously present. We explored his history, clinical course, physical examination, laboratory findings, medical treatments and actual patient condition.

**Conclusion:**

The possibilities for surgical evaluation are presented.

## Case presentation

The 27 year old man presented in our hospital with dyspnea, fatigue, dizziness, palpitations and central cyanosis with following personal history. He is the sixth child from six normal pregnancies. His family history was negative for congenital defects. After birth, a holosystolic murmur was heard along the left side of sternum and the diagnosis of Vitium cordis congenita was given based on physical examination, chest radiographs, and electrocardiogram in pediatric cardiology centre in Belgrade. Cardiac catheterization was proposed in that time. As a child, the patient had normal growth and mental development. During that time he had marked cyanosis right after a long physical activity. At the age of six he was revisited at the same Institute and they proposed to do the cardiac ultrasonography back in Kosovo. The cardiac ultrasonographic result at that time was the single ventricle. Urgent catheterization and adequate surgical treatment was considered however, not performed.

**Figure 1:**
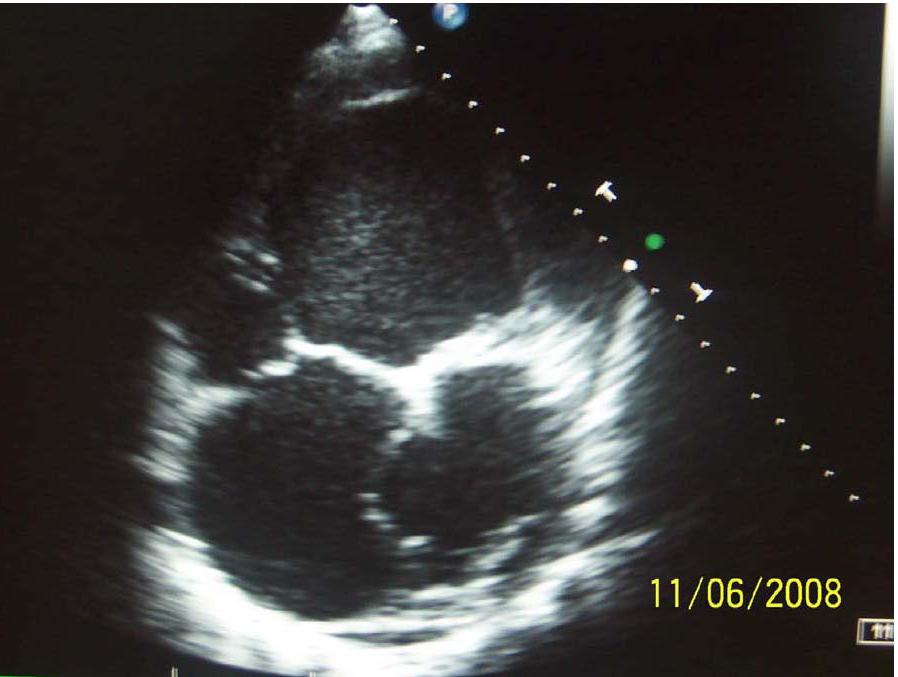
**Apical four chamber view, single ventricle interatrial aneurysm**.

The patient condition was stable with marked cyanosis just after a long physical activity until a few months ago. In our hospital he presented complaints of dyspnea, fatigue, dizziness, palpitations, and central cyanosis. In physical examination systolic murmur, tachycardia 120/min and pulmonary congestion were evident. An electrocardiogram indicated sinus tachycardia of 120/min, right axis deviation, high QRS voltage ST segment depression in inferior leads. Chest X ray signified cardiomegaly and pulmonary congestion. Transthoracic echocardiography was performed: a single ventricle, bicuspid aortic valve and aneurismatic wall of interatrial septum were seen. (Figure 1. and 2.). The transventricle diastolic diameter was 101 mm, low ejection fraction, mitral regurgitation gr. I, aortal regurgitation gr. I-II, and tricuspidal regurgitation gr. III with PSAP = 90 mmHg. Transcutaneous oxygen saturation was 80–89%. Abdominal echosonography signify considered liquid in Morrison recess. The important laboratory findings were hyperviscosity and erythrocytosis.

**Figure 2:**
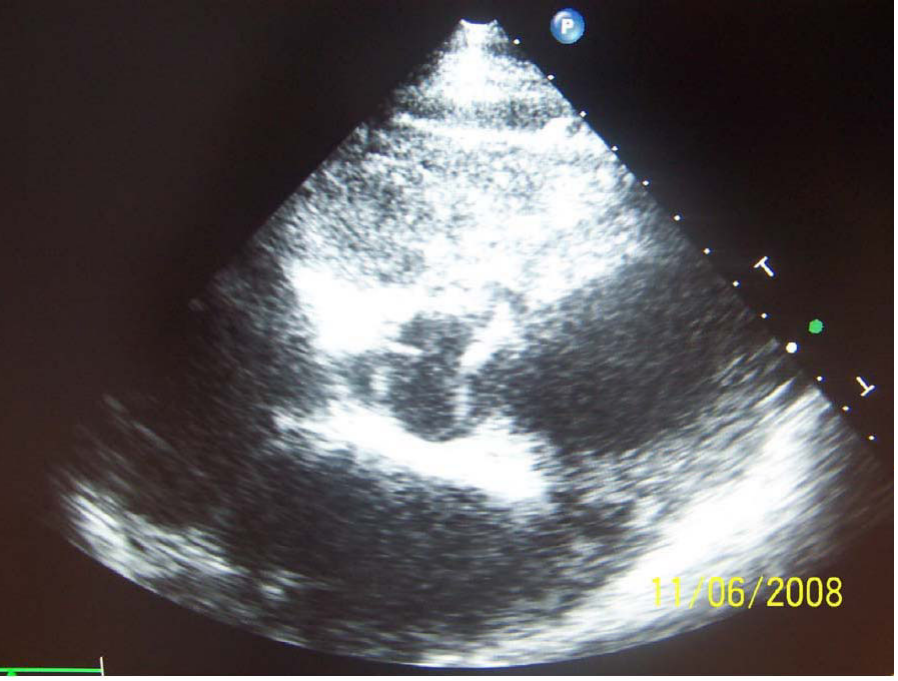
**Short axis parasternal view, bicuspid aortic valve**.

He was treated with cardiac glucozides, carvedilol, diuretics, and anticoagulants. He was discharged at stable condition with recommendation for cardiac surgery evaluation abroad since in our country it is not established yet. He couldn't get proper financial support to resolve this medical condition.

## Discussion

Anatomically, single ventricle is as a heart that is missing the smooth inflow region of an either ventricle. The atrioventricular connection in single ventricle is a double inlet or common inlet. In USA single ventricle occurs in approximately 5 of every 100.000 live births. The embryology of single ventricle is still unknown [1]. Single ventricles may be classified based on the ventricular morphology as left ventricular type (Type A:75%), right ventricular type (Type B:20%) or interdeterminate type (Type C:10–14%). In type A, transpositions of great vessels and outflow obstruction in most of cases are present. Type B is usually present with each great vessel arising from an infundibulum. In type C, aorta is usually anteriorly situated [2].

Clinical manifestations such as dyspnea, tachycardia, cyanosis and progressive heart failure are usually present after birth. Survival into late adulthood depend on presence or absence of an obstruction to the pulmonary blood flow and the pulmonary vascular resistance. In patients without pulmonary stenosis, pulmonary blood flow depends on pulmonary vascular resistance (Rp). If Rp is low, patients experience severe cardiac failure leading to death in infancy. If the patient had a Eisenmenger reaction and Rp is high, survival into adulthood is possible [3].

Optimal diastolic function is also necessary to deal with volume changes during exercise. Therefore, morphology and function of the ventricle and atrioventricular valves also are important.

Another important issue is obstruction to aortic flow. This occurs at the level of the aortic valve leading to low systemic output, hyperperfusion of the lungs and finally cardiac death.

The diagnosis may be defined by echocardiography, cardiac catheterization and cardiac magnetic resonance. Treatment in most of cases is surgical but carries a significant surgical mortality rate. These surgical treatments include shunt such as Blalock-Taussig (B-T) or Glen, placing a band on the pulmonary artery or the Fontan operation. About 65%–75% of patients without surgical corrections die during the first year of their life [4]. In this case aneurysmatic septal wall has been associated with interventricular defect. The aneurism of the interatrial septum may be in association with other congenital malformations as a foramen ovale, atrial septal defects and ventricular septal defects. Several autopsy series have reported a prevalence of 2–4% for atrial septal aneurysm (ASA). Studies performed with transesophageal echocardiography (TEE) revealed similar findings [5]. Next congenital abnormality in this case is a bicuspid aortic valve. Anatomically valve leaflet orientation can vary. Antero-posterior orientation of the commisures occurs in approximately 50–60% of cases, and horizontal orientation of the commisures is observed in 30–45% of cases [6]. Bicuspid aortic valve is the most common congenital cardiovascular anomaly, may be present in up to 1–2% of population and it may have an autosomal dominant inheritance [6,7]. Most of cases of bicuspid aortic valve are sporadic, but sometimes it may be present together with any congenital syndromes (Williams syndrome, Turner syndrome and Erdheim cystic medial necrosis) [6]. Patient with mild to moderate valve dysfunction and normal left ventricular dimensions and functions should be monitored by echocardiography at regular intervals. Aortic valve replacement is indicated for severe valve dysfunction [8].

We presented a very rare case, with single ventricle type B, bicuspid aortic valve with anterio-posterior orientation of the comisures and aneurismatic interatrial wall which has never been treated surgically and clinical consequences are seriously presented at age 27. This case is unique in medical literature. In this patient also pulmonary hypertension is present. Patients with true Eisenmenger syndrome are considered inoperable [9].

In some opinions these patients should be followed and treated conservatively [9,3].

Heart and lung transplantation is probably the only treatment option available however, with considerable morbidity and mortality in this age group therefore, it should be avoided [3].

## Consent

Written informed consent was obtained from the patient for publication of this case report and accompanying images. A copy of the written consent is available for review by the Editor-in-Chief of this journal.

## Competing interests

The authors declare that they have no competing interests.

## Authors' contributions

BB analyzed and interpreted the patient data and was a major contributor in writing the manuscript. XK analyzed and interpreted the patient data and contributed in writing the manuscript. AT analyzed and interpreted the patient data. MG analyzed the data and contributed in writing the manuscript. DK performed the echocardiography examination. All authors read and approved the final manuscript.
